# Role of lysine residues of the *Magnaporthe oryzae* effector AvrPiz‐t in effector‐ and PAMP‐triggered immunity

**DOI:** 10.1111/mpp.12779

**Published:** 2019-02-08

**Authors:** Pengfei Bai, Chan‐Ho Park, Gautam Shirsekar, Pattavipha Songkumarn, Maria Bellizzi, Guo‐Liang Wang

**Affiliations:** ^1^ Department of Plant Pathology Ohio State University Columbus OH 43210; ^2^ State Laboratory for Biology of Plant Diseases and Insect Pests, Institute of Plant Protection Chinese Academy of Agricultural Sciences Beijing 100193 China

**Keywords:** effector, lysine residue, protein stability, reactive oxygen species, rice immunity

## Abstract

*Magnaporthe oryzae *is an important fungal pathogen of both rice and wheat. However, how *M. oryzae* effectors modulate plant immunity is not fully understood. Previous studies have shown that the *M. oryzae *effector AvrPiz‐t targets the host ubiquitin‐proteasome system to manipulate plant defence. In return, two rice ubiquitin E3 ligases, APIP6 and APIP10, ubiquitinate AvrPiz‐t for degradation. To determine how lysine residues contribute to the stability and function of AvrPiz‐t, we generated double (K1,2R‐AvrPiz‐t), triple (K1,2,3R‐AvrPiz‐t) and *l*ysine‐*f*ree (LF‐AvrPiz‐t) mutants by mutating lysines into arginines in AvrPiz‐t. LF‐AvrPiz‐t showed the highest protein accumulation when transiently expressed in rice protoplasts. When co‐expressed with APIP10 in *Nicotiana benthamiana*, LF‐AvrPiz‐t was more stable than AvrPiz‐t and was less able to degrade APIP10. The avirulence of *LF‐AvrPiz‐t* on *Piz‐t:HA* plants was less than that of *AvrPiz‐t, *which led to resistance reduction and lower accumulation of the Piz‐t:HA protein after inoculation with the *LF‐AvrPiz‐t*‐carrying isolate. Chitin‐ and flg22‐induced production of reactive oxygen species (ROS) was higher in *LF‐AvrPiz‐t* than in *AvrPiz‐t* transgenic plants. In addition, *LF‐AvrPiz‐t* transgenic plants were less susceptible than *AvrPiz‐t* transgenic plants to a virulent isolate. Furthermore, both AvrPiz‐t and LF‐AvrPiz‐t interacted with OsRac1, but the suppression of OsRac1‐mediated ROS generation by LF‐AvrPiz‐t was significantly lower than that by AvrPiz‐t. Together, these results suggest that the lysine residues of AvrPiz‐t are required for its avirulence and virulence functions in rice.

## Introduction

Plant defence is a multi‐layered immune network consisting of pathogen‐associated molecular pattern (PAMP)‐triggered immunity (PTI) and effector‐triggered immunity (ETI). It relies on strategies involving a range of spatiotemporally regulated plasma membrane receptors (referred to as pattern recognition receptors, PRRs) or structurally conserved nucleotide‐binding and leucine‐rich repeat (NB‐LRR) proteins (Bent and Mackey, 2007; Jones and Dangl, 2006; Jones *et al.*, 2016). The recognition of microbial PAMPs by plant PRRs leads to the first layer of defence, PTI, which confers non‐specific resistance against pathogen infection (Boller and Felix, 2009; Zipfel, 2008). Although pathogens can evade PTI by secreting effectors into plant cells, the host may possess specific NB‐LRR proteins that sense these virulence factors, resulting in a race‐specific immune response, called ETI (Białas *et al.*, 2017; Eitas and Dangl, 2010).

The plant ubiquitination system is involved in a variety of biological processes and is essential for immunity (Banfield, 2015; Li *et al.*, 2014). It regulates one of the most important post‐translational modifications, in which substrate proteins are covalently linked to a ubiquitin chain via the lysine residues and are subjected to 26S proteasome‐dependent degradation (Ciechanover, 1998; Santner and Estelle, 2010). By exploiting the host ubiquitination machinery, pathogen effectors modify immune‐related proteins by altering their stability, enzymatic activity, relocalization or interaction with other proteins (Angot *et al.*, 2007; Spallek *et al.*, 2009). *Pseudomonas syringae *HopM1, for example, suppresses *Arabidopsis* PTI by destabilizing the immunity‐related proteins AtMIN2, AtMIN7 and AtMIN10 via the 26S proteasome degradation system (Nomura *et al.*, 2006, 2011). The RXLR‐type effector Avr3a from *Phytophthora infestans* stabilizes the host E3 ligase CMPG1 from 26S proteasome‐dependent degradation and thereby prevents CMPG‐activated host cell death during biotrophic colonization (Bos *et al.*, 2010). Instead of reprogramming host proteins for degradation, some pathogen effectors can mimic host E3 ligases to ubiquitinate and degrade target proteins. The *P. syringae *effector AvrPtoB, for example, harbours a C‐terminal E3 ligase domain that can suppress programmed cell death (PCD) (Abramovitch *et al.*, 2006; Janjusevic *et al.*, 2006). By interacting and ubiquitinating immunity‐related proteins, such as Fen (Ntoukakis *et al.*, 2009; Rosebrock *et al.*, 2007), FLS2 (Göhre *et al.*, 2008), CERK1 (Gimenez‐Ibanez *et al.*, 2009) and NPR1 (Chen *et al.*, 2017), AvrPtoB suppresses plant PTI and systemic acquired resistance (SAR) through several signalling pathways.

The importance of the plant ubiquitination system in plant–pathogen interactions has been highlighted further by the discovery of the rice blast effector AvrPiz‐t of *Magnaporthe oryzae* (Li *et al.*, 2009), which targets two rice RING‐type E3 ligases, APIP6 and APIP10 (Park *et al.*, 2012, 2016). AvrPiz‐t can be translocated into a plant cell, where it interacts with APIP6 and APIP10, and promotes their degradation via the 26S proteasome system. Silencing of either *APIP6* or *APIP10* in Nipponbare plants (NPB, with a non‐functional *Piz‐t*) reduces the PTI response, confirming that APIP6 and APIP10 are positive regulators of PTI. However, the silencing of *APIP10* in NPB‐*Piz‐t:HA* transgenic rice (NPB wild‐type plants harbouring a functional *Piz‐t:HA* gene) causes spontaneous cell death and activates *R *gene‐mediated resistance, which suggests that APIP10 acts as a negative regulator of ETI. The dual function of APIP10 suggests that AvrPiz‐t achieves its virulence and avirulence function through APIP10. By acting as the substrate of APIP10, AvrPiz‐t was ubiquitinated *in vitro* and was degraded through the 26S proteasome system in *Nicotiana benthamiana *(Park *et al.*, 2016). How the proteasomal degradation of AvrPiz‐t affects its avirulence and virulence function in rice cells is unknown.

To reveal the role of AvrPiz‐t lysine residues in ETI and PTI, we generated a *l*ysine‐*f*ree AvrPiz‐t mutant (LF‐AvrPiz‐t) and tested its avirulence and virulence functions in this study. We found that the lysine residues in AvrPiz‐t are critical for the stability of both AvrPiz‐t and APIP10 and are important for the avirulence function of AvrPiz‐t on Piz‐t and on Piz‐t accumulation during infection. In addition, the lysine residues affect the suppression of reactive oxygen species (ROS) generation triggered by PAMPs and OsRac1. Together, our results show that the lysine residues of AvrPiz‐t are required for its function in both *Piz‐t*‐mediated resistance and basal defence in rice.

## Results

### The lysine residues of AvrPiz‐t affect its protein stability in plant cells

**Figure 1:**
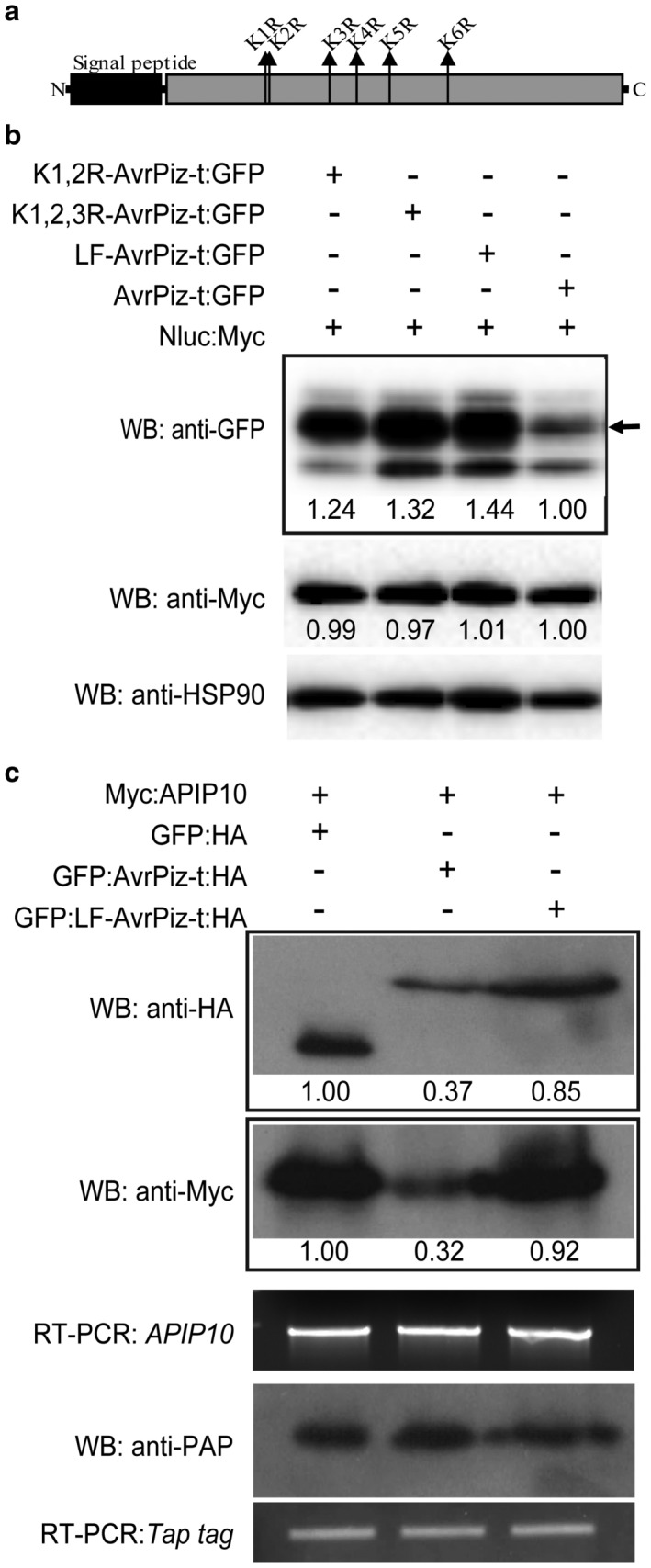
Protein stability of double, triple and lysine‐free mutants of AvrPiz‐t and APIP10 in plant cells. (a) Schematic structure of the AvrPiz‐t protein; the locations of the six lysine residues are labelled as K1–K6 according to their order in the AvrPiz‐t protein. (b) Plasmids of *K1,2R‐AvrPiz‐t:GFP*, *K1,2,3R‐AvrPiz‐t:GFP*, *LF‐AvrPiz‐t:GFP* and *AvrPiz‐t:GFP* were separately expressed in Nipponbare rice protoplasts, and *Nluc:Myc* was used as an internal transfection control. Immunoblots with anti‐GFP, anti‐Myc and anti‐HSP90 were conducted to detect the protein levels of different AvrPiz‐t mutants, Nluc‐Myc and endogenous rice HSP90, respectively. (c) Co‐expression of *Myc:APIP10* with *GFP:LF‐AvrPiz‐t:HA* and *GFP:AvrPiz‐t:HA* in *Nicotiana benthamiana*. *GFP:HA* was used as the infiltration control, and the *Tap* tag was used as an internal control. Semi‐real‐time polymerase chain reaction (semi‐RT‐PCR) was conducted for *APIP10* and *Tap* transcripts. Protein levels were measured by Image Lab software on the basis of the band intensity and were then normalized with the internal control.

As lysine residue modification affects target proteins by changing their stability, enzymatic activities, cellular translocation and protein–protein interaction (Ciechanover, 1998; Walsh *et al.*, 2005), we hypothesized that the lysine residues of AvrPiz‐t, as the potential ubiquitin acceptor sites, are essential for AvrPiz‐t stability and function. As shown in Fig. 1a, AvrPiz‐t contains six lysine residues. To determine the effect of the lysine residues, we used a site‐directed mutagenesis method to generate a series of AvrPiz‐t mutants harbouring double (K1,2R‐AvrPiz‐t), triple (K1,2,3R‐AvrPiz‐t) and all six (LF‐AvrPiz‐t) lysine to arginine mutations. Compared with the wild‐type AvrPiz‐t:GFP, transient expression of K1,2R‐AvrPiz‐t:GFP, K1,2,3R‐AvrPiz‐t:GFP and LF‐AvrPiz‐t:GFP showed a higher protein accumulation in rice protoplasts (Fig. 1b). Among them, LF‐AvrPiz‐t was the most stable in terms of protein stability, suggesting that, although the mutation of individual lysine residues can increase AvrPiz‐t protein stability, mutation of all six lysine residues can lead to maximum protein stability in rice cells. Therefore, we selected LF‐AvrPiz‐t for subsequent experiments. When co‐expressed with Myc‐tagged E3 ligase APIP10 in *N. benthamiana*, the GFP:LF‐AvrPiz‐t:HA protein was more stable than the wild‐type GFP:AvrPiz‐t:HA (Fig. 1c, first panel). Meanwhile, the protein levels of Myc:APIP10 were higher when it was co‐expressed with LF‐AvrPiz‐t than when it was co‐expressed with wild‐type AvrPiz‐t (Fig. 1c, second panel). These results suggest that the lysine residues of AvrPiz‐t are essential for its stability and, consequently, for its ability to degrade APIP10 in plant cells.

### The lysine residues of AvrPiz‐t are required for the activation of *Piz‐t*‐mediated resistance

**Figure 2:**
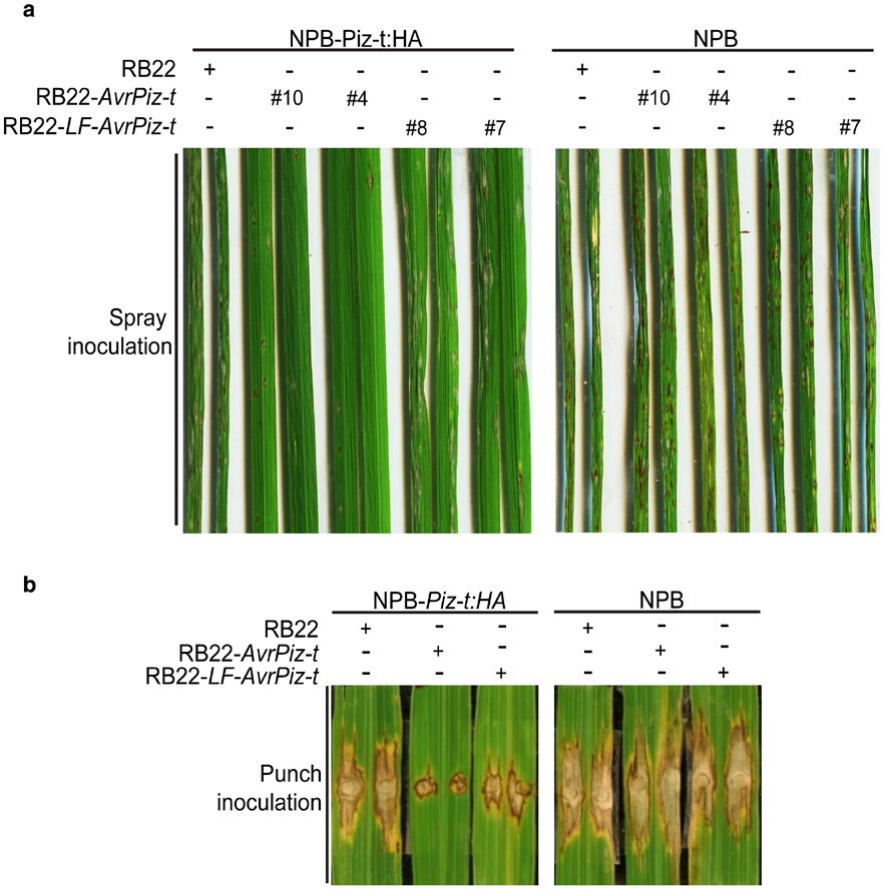
Effects of the lysine residues of AvrPiz‐t on the activation of *Piz‐t*‐mediated resistance. (a) Effects of spray inoculation of NPB‐*Piz‐t:HA* transgenic rice and NPB wild‐type rice with different *Magnaporthe oryzae* RB22 isolates. Two transformants of the *M. oryzae* isolate RB22 harbouring the *LF‐AvrPiz‐t* or *AvrPiz‐t* constructs were used for inoculation, whereas the non‐transformed *M. oryzae* RB22 was used as a control. Infected leaves were assessed at 6 days post‐inoculation (dpi). (b) Effects of punch inoculation of NPB‐*Piz‐t:HA* transgenic rice and NPB wild‐type rice with different *M. oryzae* RB22 isolates. *Magnaporthe oryzae* RB22‐*LF‐AvrPiz‐t* or RB22‐*AvrPiz‐t* transformants from the spray inoculation were used. Six‐week‐old plants were inoculated with an *M. oryzae* spore suspension. Infected leaves were assessed at 9 dpi.

To investigate the functional role of lysine residues in the recognition by Piz‐t, we generated *M. oryzae* RB22 transformants harbouring the *AvrPiz‐t* or *LF‐AvrPiz‐t* constructs under the control of the native promoter. The *M. oryzae* RB22 wild‐type isolate that lacks a functional *AvrPiz‐t* was used for fungal transformation. We obtained six independent transformants of each construct after polymerase chain reaction (PCR) screening and selected two per construct for the following experiments. We then spray inoculated NPB plants with the RB22‐*AvrPiz‐t* and RB22‐*LF‐AvrPiz‐t* transformants and found that their pathogenicity did not differ from that of the RB22 wild‐type isolate (Fig. 2a, right panel; Fig. S1, see Supporting Information). To determine whether the lysine mutations in AvrPiz‐t disrupt its recognition of Piz‐t, we spray inoculated NPB‐*Piz‐t:HA* plants with RB22‐*AvrPiz‐t* and RB22‐*LF‐AvrPiz‐t* transformants. Inoculation with RB22‐*AvrPiz‐t* resulted in a resistance phenotype with no lesions, whereas inoculation with RB22‐*LF‐AvrPiz‐t* resulted in a susceptible phenotype with merged, necrotic lesions, a phenotype similar to that resulting from inoculation with the RB22 wild‐type isolate (Fig. 2a, left panel; Fig. S1). Results with punch inoculation of NPB‐*Piz‐t:HA* plants were consistent with the results of spray inoculation, i.e. disease was more significant on plants inoculated with the RB22‐*LF‐AvrPiz‐t* transformant than with the RB22‐*AvrPiz‐t* transformant, but the lesions were smaller with the RB22‐*LF‐AvrPiz‐t* transformant than with the RB22 wild‐type (Fig. 2b). These results suggest that mutations of the lysine residues of AvrPiz‐t partially abolish its avirulence function, thus leading to the failure of *Piz‐t‐*mediated resistance.

### LF‐AvrPiz‐t fails to induce Piz‐t accumulation during *M. oryzae* infection

**Figure 3:**
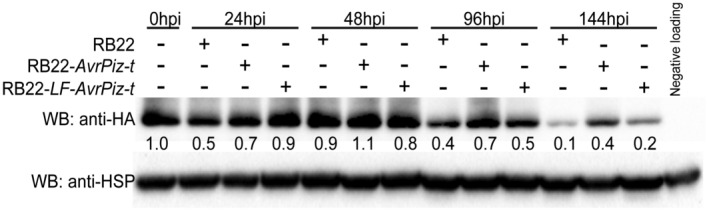
Effects of the lysine residues on AvrPiz‐t‐induced Piz‐t protein accumulation during *Magnaporthe oryzae* infection. NPB‐*Piz‐t:HA* transgenic plants were inoculated with *M. oryzae* isolate RB22, RB22‐*AvrPiz‐t* or RB22‐*LF‐AvrPiz‐t*. Immunoblots with anti‐HA were conducted to detected Piz‐t protein accumulation at different time points after inoculation (hpi, hours post‐inoculation). Endogenous rice HSP90 detected by anti‐HSP was used as an internal control, and NPB wild‐type samples served as a negative control (last lane). Protein levels were measured by Image Lab software on the basis of the band intensity and were then normalized with the internal control.

Previous studies have shown that AvrPiz‐t is translocated from *M. oryzae* into rice cells, where it induces a high level of Piz‐t protein accumulation during *M. oryzae* infection (Park *et al.*, 2012, 2016). Given the altered stability and avirulence function of LF‐AvrPiz‐t, we speculated that the lysine residue mutations of AvrPiz‐t might affect Piz‐t accumulation. To obtain a comprehensive profile of Piz‐t protein accumulation during *M. oryzae* infection, we spray inoculated NPB‐*Piz‐t:HA* transgenic rice with the *M. oryzae* RB22‐*LF‐AvrPiz‐t *transformant, the RB22‐*AvrPiz‐t *transformant and the RB22 wild‐type, respectively. Infected tissues were collected at 0, 24, 48, 96 and 144 h post‐inoculation (hpi). Immunoblot analysis showed that the protein levels of Piz‐t in RB22‐*LF‐AvrPiz‐t‐*inoculated plants were slightly higher than those in RB22‐*AvrPiz‐t*‐inoculated plants at 24 hpi (Fig. 3). However, the protein levels of Piz‐t in RB22‐*LF‐AvrPiz‐t‐*inoculated plants were lower than those in RB22‐*AvrPiz‐t‐*inoculated plants at 48, 96 and 144 hpi (Fig. 3). The profiles of Piz‐t protein accumulation were consistent with the inoculation results, confirming the importance of lysine residues for the avirulence function of AvrPiz‐t.

### The lysine residues of AvrPiz‐t are necessary for the suppression of basal resistance

**Figure 4:**
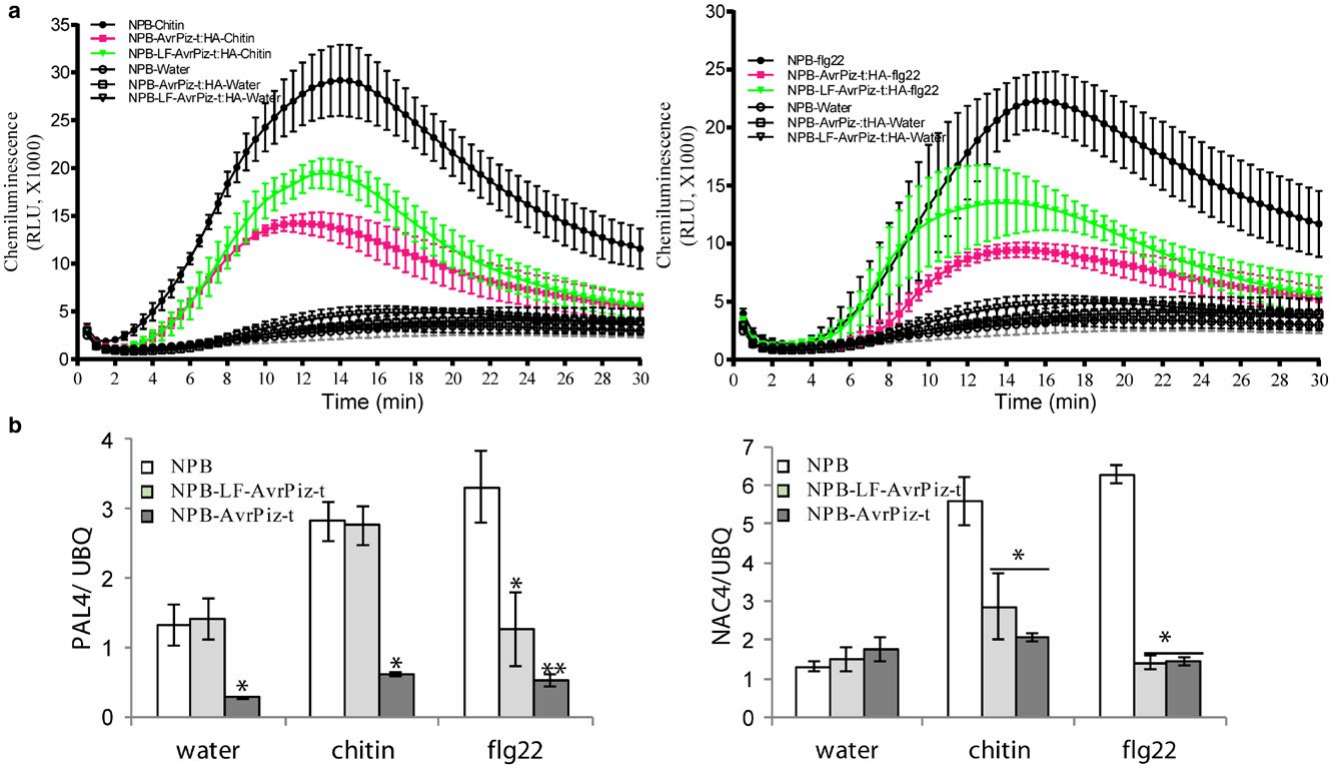
Reactive oxygen species (ROS) and defence gene expression in NPB‐*LF‐AvrPiz‐t* plants. (a) Pathogen‐associated molecular pattern (PAMP)‐induced ROS production in NPB‐*LF‐AvrPiz‐t:HA *transgenic plants, NPB‐*AvrPiz‐t:HA* transgenic plants and NPB wild‐type plants. Values are means ± standard error (SE) (*n* = 3). RLU, relative light unit. (b) PAMP‐induced marker gene expression in NPB‐*LF‐AvrPiz‐t:HA *transgenic plants, NPB‐*AvrPiz‐t:HA* transgenic plants and NPB wild‐type plants. The transcriptional levels of two defence‐related marker genes, *PAL4* and *NAC4*, at 3 h after chitin or flg22 treatment were measured by quantitative real‐time polymerase chain reaction (qRT‐PCR). Values are means ± SE (*n* = 3). One‐way analysis of variance (ANOVA) test with Tukey’s method was conducted. Significant differences at *P* < 0.05 and *P* < 0.01 are indicated by ‘*’ and ‘**’, respectively. UBQ, ubiquitin.

In the absence of Piz‐t recognition, AvrPiz‐t suppresses host PTI responses by acting as a virulence effector (Park *et al.*, 2012). To examine whether mutation of the lysine residues of AvrPiz‐t impairs its virulence function, we generated stable transgenic rice by expressing *LF‐AvrPiz‐t:HA* and *AvrPiz‐t:HA* under the maize ubiquitin promoter in NPB plants [NPB‐*LF‐AvrPiz‐t:HA* in this study and NPB‐*AvrPiz‐t:HA *in Park *et al. *(2012)]. Quantitative real‐time polymerase chain reaction (qRT‐PCR) analysis showed high transcriptional levels of both *LF‐AvrPiz‐t:HA* and *AvrPiz‐t:HA *in transgenic plants (Fig. S2, see Supporting Information). By using luminol‐based chemiluminescence, we quantified the chitin‐ and flg22‐induced ROS production in NPB‐*LF‐AvrPiz‐t:HA* and NPB‐*AvrPiz‐t:HA* transgenic plants. Consistent with the results of Park *et al. *(2012), ectopic expression of *AvrPiz‐t* in NPB plants suppressed the production of ROS elicited by chitin or flg22 treatment, but the suppression ability was partially reduced in NPB‐*LF‐AvrPiz‐t:HA* plants (Fig. 4a). In addition to the ROS response, we also measured the transcriptional changes of the plant defence marker genes *PAL4* and *NAC4* caused by chitin or flg22 treatment of NPB‐*LF‐AvrPiz‐t:HA* and NPB‐*AvrPiz‐t:HA* plants. qRT‐PCR analysis showed that the expression of *PAL4* in chitin‐treated NPB‐*LF‐AvrPiz‐t:HA* plants was completely recovered as in NPB plants, but, in flg22‐treated NPB‐*LF‐AvrPiz‐t:HA* plants, the expression of *PAL4* was only partially recovered (Fig. 4b, left panel). The expression of *NAC4*, however, did not differ significantly between NPB‐*LF‐AvrPiz‐t:HA* and NPB‐*AvrPiz‐t:HA* transgenic plants (Fig. 4b, right panel).

**Figure 5:**
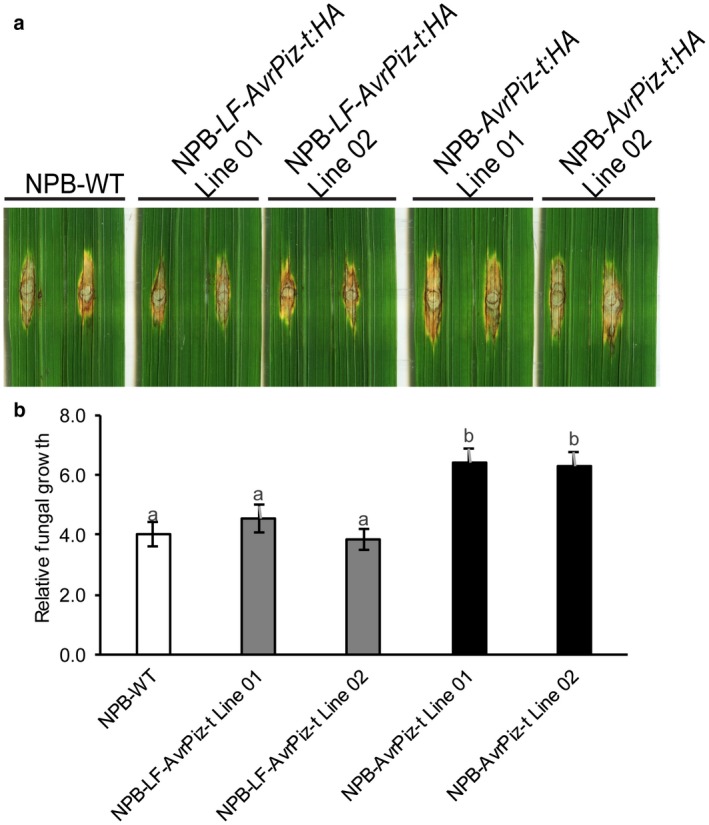
Effects of the lysine residues of AvrPiz‐t on the suppression of rice basal resistance. (a) Effects of punch inoculation of NPB‐*LF‐AvrPiz‐t:HA* and NPB‐*AvrPiz‐t:HA* transgenic plants with *Magnaporthe oryzae* isolate RB22. Two independent plants of each transgenic line were used. NPB wild‐type plants served as a control. Six‐week‐old plants were inoculated with an *M. oryzae* spore suspension of 5 × 10^5^ spores/mL. Phenotype leaves were assessed at 9 days post‐inoculation (dpi). (b) Relative fungal growth in the photographed leaves was measured by DNA‐based real‐time PCR to quantify the amount of DNA of *M. oryzae* using two sets of primers specific for *M. oryzae Pot2* and rice *Ubiquitin*. Values are means ± standard error (SE) (*n* = 3). One‐way analysis of variance (ANOVA) test with Tukey’s method was conducted. Means followed by different letters are significantly different (*P* < 0.05).

Given the differences in ROS generation and defence gene expression between NPB‐*LF‐AvrPiz‐t:HA* and NPB‐*AvrPiz‐t:HA* transgenic plants, we reasoned that the lysine residue mutations in AvrPiz‐t might result in a change in the disease phenotype of NPB‐*LF‐AvrPiz‐t:HA* plants. Using punch inoculation with the virulent isolate RB22, we quantified disease development in transgenic plants and NPB plants. On the basis of lesion size and fungal biomass, disease symptoms were fewer in NPB‐*LF‐AvrPiz‐t:HA* plants than in NPB‐*AvrPiz‐t:HA* plants (Fig. 5a,b). These results, together with the reduced ability of NPB‐*LF‐AvrPiz‐t:HA* to suppress ROS generation and *PAL4* expression, suggest that the lysine residues of AvrPiz‐t are essential for the suppression of plant basal defence.

### AvrPiz‐t interacts with Rac1 and suppresses Rac1‐mediated ROS accumulation in *N. benthamiana*, and lysine residues are required for this suppression

**Figure 6:**
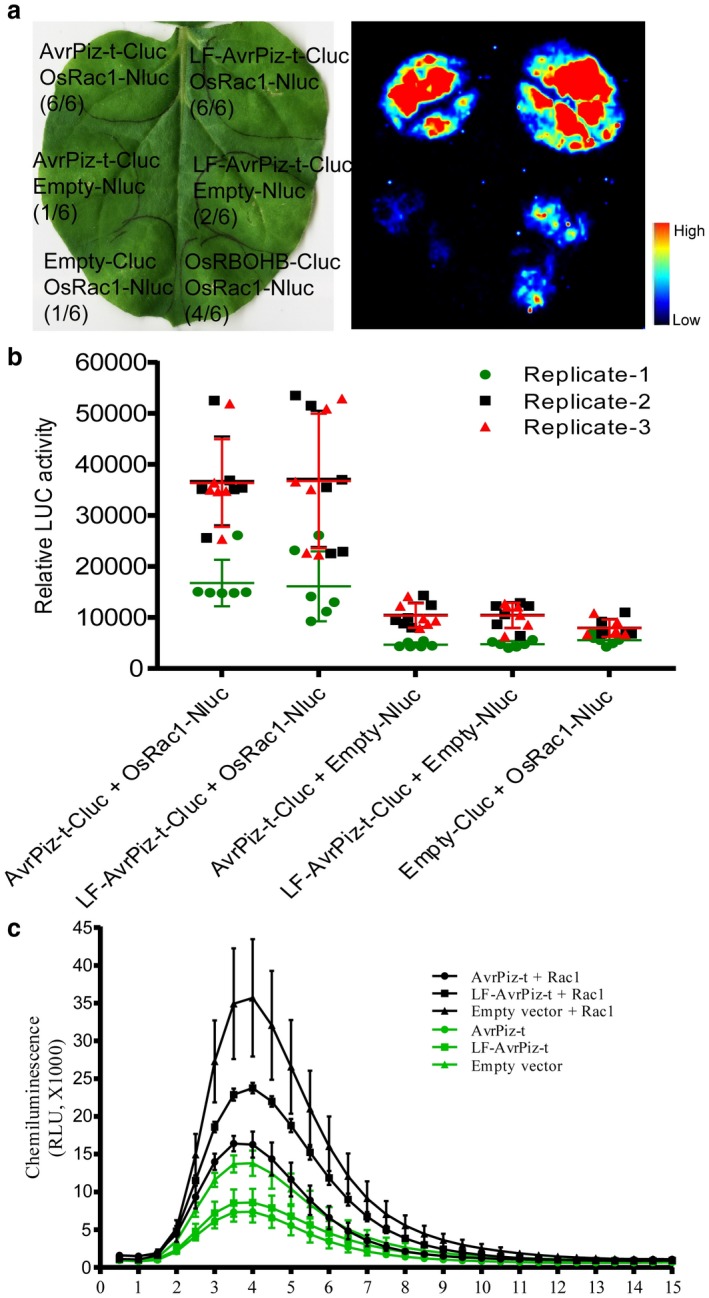
Interaction between OsRac1 and AvrPiz‐t/LF‐AvrPiz‐t and suppression of Rac1‐mediated reactive oxygen species (ROS) accumulation by AvrPiz‐t and LF‐AvrPiz‐t in *Nicotiana benthamiana*. (a) Luciferase complementation imaging (LCI) assay for the interaction between OsRac1 and AvrPiz‐t/LF‐AvrPiz‐t. *OsRac1‐Nluc* was co‐expressed with *AvrPiz‐t‐Cluc* or *LF‐AvrPiz‐t‐Cluc*, whereas *Nluc*‐empty vector/*AvrPiz‐t‐Cluc*, *Nluc*‐empty vector/*LF‐AvrPiz‐t‐Cluc* and *OsRac1‐Nluc*/*Cluc*‐empty vector were used as negative controls. *OsRac1‐Nluc*/*OsRBOHB‐Cluc* was used as a positive control. The numbers in parentheses indicate the number of leaf spots with positive luminescence signals among all of the infiltrated leaves. (b) Relative luciferase (LUC) activity of the interaction between OsRac1 and AvrPiz‐t/LF‐AvrPiz‐t. Three biological replicates with 18 *N. benthamiana* leaves were measured for the luciferase activity using the Image Lab quantity tool. (c) Rac1‐mediated ROS production in *N. benthamiana* leaves expressing *AvrPiz‐t*, *LF‐AvrPiz‐t* or empty vector with *OsRac1*. Leaf discs of each infiltrated spot on the same leaves were treated with 8 nm chitin. ROS accumulation was measured using luminol‐based chemiluminescence for 15 min. Values are means ± standard error (SE) (*n* = 3). RLU, relative light unit.

OsRac1, a rice homologue of human small GTPase, is an essential regulator of defence‐related ROS production and cell death in rice (Kawasaki *et al.*, 1999; Ono *et al.*, 2001). With the hypothesis that OsRac1 might act as an effector target used by the pathogen to manipulate host ROS production, we tested the interaction between OsRac1 and AvrPiz‐t using the luciferase complementation imaging (LCI) assay. Strong luminescence signals were observed for the interactions of OsRac1 with both AvrPiz‐t and LF‐AvrPiz‐t, respectively (Fig. 6a). The average relative luciferase activities from three replications of the AvrPiz‐t/OsRac1 and LF‐AvrPiz‐t/OsRac1 co‐infiltrations were similar to each other, but higher than the negative controls (Fig. 6b), suggesting that lysine mutations do not affect the AvrPiz‐t and OsRac1 interaction.

To assess whether AvrPiz‐t interferes with OsRac1‐mediated ROS accumulation and whether the lysine residues in AvrPiz‐t are required for this interference, we co‐expressed *OsRac1* and *AvrPiz‐t/LF‐AvrPiz‐t* in *N. benthamiana* and measured chitin‐induced ROS production by luminol‐based chemiluminescence. *AvrPiz‐t/LF*, *AvrPiz‐t* and the empty vector (EV) were agroinfiltrated 1 day before *OsRac1* in the same leaves. Consistent with the reduced ROS induction in NPB‐*AvrPiz‐t:HA* transgenic rice (Fig. 4a), the levels of ROS accumulation were suppressed by transient expression of either *AvrPiz‐t* or *LF‐AvrPiz‐t* in *N. benthamiana* (Fig. 6c). However, the suppressive ability of LF‐AvrPiz‐t was partially impaired. These results suggest that AvrPiz‐t suppresses OsRac1‐mediated ROS production *in planta* and that the lysine residues of AvrPiz‐t are required for this suppression.

## Discussion

Pathogen effectors are essential for the infection of host plants. Although many effectors have been characterized in different pathogens, little is known about the role of individual domains or amino acids in effector functions because most effectors lack a conserved motif or domain. AvrPiz‐t is a small secreted peptide with 108 predicted amino acids (Li *et al.*, 2009). Structural analysis has revealed that two cysteine residues, Cys62 and Cys75, in AvrPiz‐t form a disulfide bond, which is responsible for its avirulence function (Zhang *et al.*, 2013). Our previous studies have shown that AvrPiz‐t is ubiquitinated by two rice E3 ligases, APIP6 and APIP10 (Park *et al.*, 2012, 2016). The role of the lysine residues of AvrPiz‐t in APIP6/APIP10‐mediated ubiquitination, and its interaction with host immunity, is unclear. In this study, we used genetic, biochemical, and transient and stable plant expression approaches to determine the function of lysine residues in AvrPiz‐t. Transient expression of *LF‐AvrPiz‐t* *in planta *increases its protein accumulation, suggesting that the lysine residues of AvrPiz‐t are essential for the stability of the protein. It is possible that LF‐AvrPiz‐t shows lesser binding affinity to APIP6, APIP10 or other unknown host proteins, thus reducing the degradation rate in rice cells. Another possibility is that the protein folding change in LF‐AvrPiz‐t increases its stability *in planta*.

The importance of lysine residues in AvrPiz‐t is also indicated by the reduced ability of LF‐AvrPiz‐t to degrade APIP10 relative to the wild‐type AvrPiz‐t in *N. benthamiana*. Our previous study demonstrated how APIP10 connects AvrPiz‐t to its cognate Piz‐t for immune activation (Park *et al.*, 2016). In the absence of pathogen invasion, APIP10 maintains the homeostasis of the R protein Piz‐t at an optimal level by the 26S proteasome degradation system. Once AvrPiz‐t is translocated into rice cells during pathogen infection, AvrPiz‐t promotes the degradation of APIP10, thus removing the suppression effects of APIP10 on the accumulation of Piz‐t. In this study, we found that the RB22‐*LF‐AvrPiz‐t* isolate failed to cause Piz‐t accumulation and *Piz‐t*‐mediated resistance. These results demonstrate that the lysine residues in AvrPiz‐t are required for the activation of *Piz‐t*‐mediated resistance to *M. oryzae*. However, how the reduction in APIP10 levels by LF‐AvrPiz‐t affects Piz‐t accumulation remains to be elucidated.

In addition to failing to activate ETI, LF‐AvrPiz‐t partially lost its virulence function in the suppression of PTI responses. Previous research has reported that many microbial effectors can suppress PTI and, in some cases, suppression results from reprogramming of the fate of basal defence‐related proteins (Banfield, 2015; Duplan and Rivas, 2014). AvrPiz‐t interferes with the ubiquitination activities of the rice E3 ligase proteins APIP6 and APIP10, which are positive regulators of PTI in the non‐*Piz‐t* background. In this study, susceptibility to *M. oryzae* was lower with the overexpression of *LF‐AvrPiz‐t* than with the overexpression of *AvrPiz‐t* in the NPB wild‐type, which is consistent with the impaired ability of LF‐AvrPiz‐t to suppress ROS production and defence‐related gene expression in response to PAMP treatments. These results suggest that the lysine residues of AvrPiz‐t are essential for its virulence function, and that the underlying mechanism could involve reduced interference of APIP6 or APIP10 activity by LF‐AvrPiz‐t. Because LF‐AvrPiz‐t retains some ability to suppress PTI, it is likely that LF‐AvrPiz‐t retains part of its virulence activity without these residues.

The ROS burst is a hallmark of plant defence which is associated with PCD and defence activation. OsRac1 is activated for chitin‐triggered ROS production by interaction with the plasma membrane complex OsCEBiP–OsCERK1–OsRacGEF1 (Akamatsu *et al.*, 2013). We demonstrated that OsRac1 interacts with both AvrPiz‐t and LF‐AvrPiz‐t, suggesting that OaRac1 might serve as an effector target for the manipulation of ROS production in host cells. Previous studies have shown that AvrPiz‐t suppresses BAX‐induced PCD in *N. benthamiana* (Li *et al.*, 2009) and inhibits the transcriptional activity of a bZIP transcriptional factor APIP5, thus enhancing APIP5‐mediated necrosis, which is beneficial for the necrotrophic growth of *M. oryzae *in rice cells (Li *et al.*, 2009; Wang *et al.*, 2016). The involvement of AvrPiz‐t in cell death suggests that it could play a role in the regulation of OsRac1‐mediated ROS production and defence activation. It will be interesting to determine whether the lysine mutations in AvrPiz‐t affect OsRac1 activity or its interaction with the defence complex OsCEBiP–OsCERK1–OsRacGEF1 in the future.

## Experimental Procedures

### Rice cultivation, *M. oryzae* inoculation and disease evaluation

The rice plants used in this study were germinated on half‐strength Murashige and Skoog (½MS) medium (24 h light at 25 °C) for 1 week, and seedlings of a uniform size were transplanted into soil. For germination, rice seeds with removed husks were sterilized by immersion in 75% ethanol for 1 min and then in 2% sodium hypochlorite (Clorox bleach) for 30 min (Park *et al.*, 2012). After they had been rinsed with sterile water, the seeds were placed on ½MS medium and kept at 25 °C with 24 h light for 1 week. Seedlings of a uniform size were then transplanted into soil. Plants used for fungal inoculation or ROS assays were kept in a growth chamber (Conviron, model BDR16, Winnipeg, Canada) with 12 h of light at 26 °C, 12 h of darkness at 20 °C and 80% relative humidity.

The *M. oryzae* isolates were cultured on oatmeal agar medium (30 g of ground Quaker Oats oatmeal, 15 g of agar, increased to 1 l with water) in a dark, 28 °C incubator for 1 week, and were then exposed to fluorescent light in a 28 °C incubator for a further week. The first week in the dark supported hyphal growth, and the second week with fluorescent light supported sporulation. For the standardized inoculation of rice with *M. oryzae* (Ono *et al.*, 2001), the conidia of *M. oryzae* isolates were collected (0.1% Tween‐20) and their concentration was adjusted with a haemocytometer to 2 × 10^5^ spores/mL for spray inoculation or to 5 × 10^5^ spores/mL for punch inoculation.

For spray inoculation (Park *et al.*, 2012; Qu *et al.*, 2006), 3‐week‐old rice plants were placed in a plastic bag (to provide high humidity) and the leaves were evenly sprayed with fungal spore suspensions. Spray‐inoculated plants were kept in the plastic bag in the dark for 24 h before they were returned to the growth chamber. Six days after spray inoculation, disease reactions of inoculated plants were scored using a 0–5 scale, where ‘0’ represents a resistance phenotype with no lesions and ‘5’ represents a susceptible phenotype with coalesced lesions. For punch inoculation, 6‐week‐old plants were used. The second leaf from the top of each plant was wounded, but not cut through, with a mouse ear punch, and the wound was then inoculated with a droplet (10 µL) of a spore suspension. The inoculated wound was wrapped with Scotch tape so that the droplet remained on the wound. The inoculated leaves were photographed at 9 days post‐inoculation (dpi) and then subjected to cetyltrimethylammonium bromide (CTAB)‐based DNA extraction (Murray and Thompson, 1980). To quantify the relative fungal growth, the ratio of fungal DNA to plant DNA was determined by qRT‐PCR as described previously (Park *et al.*, 2012).

### Rice and *M. oryzae* transformation

*Agrobacterium*‐mediated rice transformations for NPB‐*LF‐AvrPiz‐t:HA* and NPB‐*AvrPiz‐t:HA* overexpression plants were conducted with the modified protocol as described previously (Hiei *et al.*, 1994; Nishimura *et al.*, 2006). Rice embryonic calli induced from mature seeds of NPB rice were co‐cultivated with agrobacteria LBA4404 harbouring the pCXUN‐*LF‐AvrPiz‐t:HA* construct or the pCXUN‐*AvrPiz‐t:HA* construct. Positive transformants were selected on hygromycin medium and transferred onto regenerating medium for plantlet formation. To obtain homozygous lines, we selected the next generation of transgenic plants on hygromycin medium and performed a PCR genotyping. Polyethylene glycol (PEG)‐mediated *M. oryzae* transformations for RB22‐*LF‐AvrPiz‐t:HA* and RB22‐*AvrPiz‐t:HA *isolates were generated as described previously (Shirsekar, 2013). *Magnaporthe oryzae* isolate RB22, which lacks a functional *AvrPiz‐t*, was used for the fungal transformations.

### Rice protoplast isolation and transfection

Rice protoplasts were isolated and transfected by following published protocols with minor modification (Yoo *et al.*, 2007; Zhang *et al.*, 2011). Ten‐day‐old etiolated rice seedlings were grown on ½MS medium in the dark at 28 °C. The sheath and stem parts of the seedlings were cut into 0.5‐mm fragments with a razor blade and immediately submerged into cell wall digestion buffer [1.5% Cellulase RS, 0.75% Macerozyme R‐10, 0.6 m mannitol, 10 mm 2‐(N‐Morpholino)ethanesulfonic acid hydrate, 4‐Morpholineethanesulfonic acid (MES) (Sigma Cat. No. 2933, St. Louis, MO, USA), 10 mm CaCl_2_ and 0.1% bovine serum albumin (BSA), pH 5.7]. The digestion was conducted under vacuum infiltration (20 kPa) for 30 min, and the digested tissue was then incubated for another 5–6 h in the dark with gentle shaking (60–80 rpm). After digestion, rice residues were washed three times with W5 solution (154 mm NaCl, 125 mm CaCl_2_, 5 mm KCl, 2 mm MES and 0.5% glucose, pH 5.7) and filtered through 40‐μm nylon mesh. Protoplasts were harvested by centrifugation of the filtered solution at 1500 rpm for 3 min. The pelleted protoplasts were washed three times with W5 solution and then resuspended in MMG solution (0.4 m mannitol, 15 mm MgCl_2_ and 4 mm MES, pH 5.7) for transfection. PEG‐mediated protoplast transfections were conducted using 200 μL of protoplasts per 5 μg of plasmids in a 2‐mL microfuge tube. An equal volume of PEG solution [40% (w/v) PEG 4000 (Sigma Cat. No. 81240), 0.2 m mannitol and 0.1 m CaCl_2_] was added and mixed well. The mixture was incubated at room temperature for 15 min in the dark. Then, two volumes of W5 solution were added to terminate PEG effects. Protoplasts were collected by centrifugation, and the pellet was resuspended in W5 solution and incubated in the dark for 16 h.

### Luciferase complementation imaging assay

*In vivo* protein–protein interactions were detected via luciferase complementation imaging assay, as described previously (Chen *et al.*, 2008). OsRac1 was fused with N‐terminal luciferase (Nluc), whereas LF‐AvrPiz‐t and AvrPiz‐t were fused with C‐terminal luciferase (Cluc). *Agrobacterium* strain GV3101, carrying *Nluc* or *Cluc* constructs, was mixed and infiltrated into leaves of *N. benthamiana*. The pair of OsRac1 and OsRBOHB served as a positive interaction control. Three days after agroinfiltration, leaves co‐expressing different constructs were treated with 1 mm luciferin substrate and subjected to luminescence intensity measurement via a Bio‐Rad ChemiDoc XRS+ imaging system (Bio‐Rad, Hercules, CA, USA).

### ROS quantification by luminol‐based chemiluminescence

A luminol‐based chemiluminescence method was used to quantify ROS accumulation in rice and *N. benthamiana* leaves in response to PAMP treatment (Schwacke and Hager, 1992; Smith and Heese, 2014). In brief, discs of 6‐week‐old rice leaves (the third leaf from the top) or of agroinfiltrated *N. benthamiana* leaves were placed in sterile water overnight and then submerged in reaction mixture [100 µL of Immunstar‐HRP substrate from Bio‐Rad (Cat. No. 170‐5040) and 1 µL of peroxidase‐streptavidin from Jackson Immunoresearch (Cat. No. 016‐030‐084)] with 8 nm chitin (Toronto Research Chemicals, Cat. No. H290750, *N*,*N*′,*N*′′,*N*′′′,*N*′′′′,*N*′′′′′‐hexaacetylchitohexaose, Toronto, Canada) or 100 nm flg22. All reaction mixtures were immediately subjected to luminescence measurement in a Glomax 20/20 Luminometer (one measurement per 10 s for 15–30 min) (Madison, WI, USA).

### qRT‐PCR analysis

To quantify the expression levels of marker genes (*PAL4* and *NAC4*) after PAMP treatments, leaf discs of different transgenic rice plants were immersed in water containing either 100 nm flg22 or 8 nm chitin for 3 h, as described previously (Park *et al.*, 2012). Total RNA was extracted with Trizol reagent (Invitrogen, Cat. No. 15596, Carlsbad, CA, USA) and subjected to first‐strand cDNA synthesis using a Promega RT‐PCR kit (Madison, WI, USA). qRT‐PCR was performed using an iQ5 real‐time PCR detection system (Bio‐Rad). The transcriptional levels were calculated by the relative 2^−ΔCT^ method with the rice ubiquitin gene as an endogenous reference for normalization.

### Constructs and primer sequences used in this study

The plasmid constructs, primers and their purposes are listed in Table S1 (see Supporting Information). The primer sequences for qRT‐PCR are listed in Table S2 (see Supporting Information).

## Supporting information


**Fig. S1  **Rice blast scores of the NPB‐*Piz‐t:HA* and NPB plants spray inoculated with the *AvrPiz‐t‐* and *LF‐AvrPiz‐t*‐carrying isolates of *Magnaporthe oryzae*. The phenotype results are presented in Fig. 2a. Disease reactions of 30 seedlings per isolate were scored using a 0–5 scale system. Infected leaves were photographed at 6 days post‐inoculation (dpi) as shown in Fig. 2. Values are means ± standard error (SE) (*n* = 30). One‐way analysis of variance (ANOVA) test with Tukey's method was conducted. Means with different letters are significantly different (*P* < 0.05).

**Fig. S2  **Transcriptional expression of *LF‐AvrPiz‐t:HA* and *AvrPiz‐t:HA* in transgenic rice. Leaf tissues of the transgenic lines used for punch inoculation were sampled before inoculation. The quantitative real‐time polymerase chain reaction (qRT‐PCR) analysis was conducted with the gene‐specific primers to both *LF‐AvrPiz‐t:HA* and *AvrPiz‐t:HA*. Values are means ± standard error (SE) (*n* = 30). One‐way analysis of variance (ANOVA) test with Tukey's method was conducted. Means with different letters are significantly different (*P* < 0.05).

**Table S1  **List of plasmid constructs and primers used in this study.

**Table S2  **List of primers used for quantitative real‐time polymerase chain reaction (qRT‐PCR).
