# Supplementary figures and images for: Role of lysine residues of the Magnaporthe oryzae effector AvrPiz‐t in effector‐ and PAMP‐triggered immunity
Source: Mol Plant Pathol. 2019 Feb 8;20(4):599–608. doi: 10.1111/mpp.12779 (PMC6637882; doi:10.1111/mpp.12779)

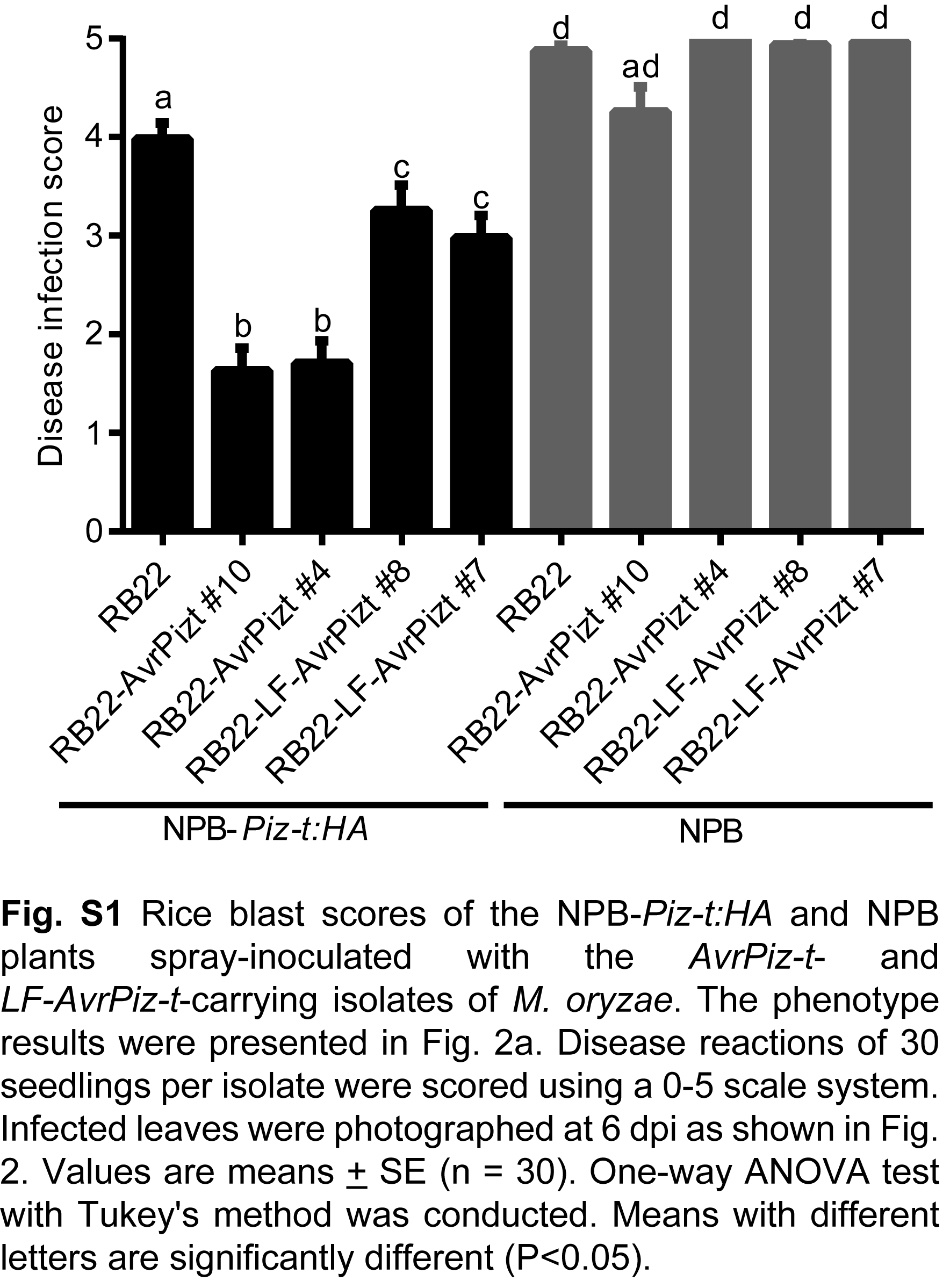

Supplement: Supplementary file 1 — Fig. S1 Rice blast scores of the NPB‐Piz‐t:HA and NPB plants spray inoculated with the AvrPiz‐t‐ and LF‐AvrPiz‐t‐carrying isolates of Magnaporthe oryzae. The phenotype results are presented in Fig. 2a. Disease reactions of 30 seedlings per isolate were scored using a 0–5 scale system. Infected leaves were photographed at 6 days post‐inoculation (dpi) as shown in Fig. 2. Values are means ± standard error (SE) (n = 30). One‐way analysis of variance (ANOVA) test with Tukey's method was conducted. Means with different letters are significantly different (P < 0.05). [file MPP-20-599-s001.tif]

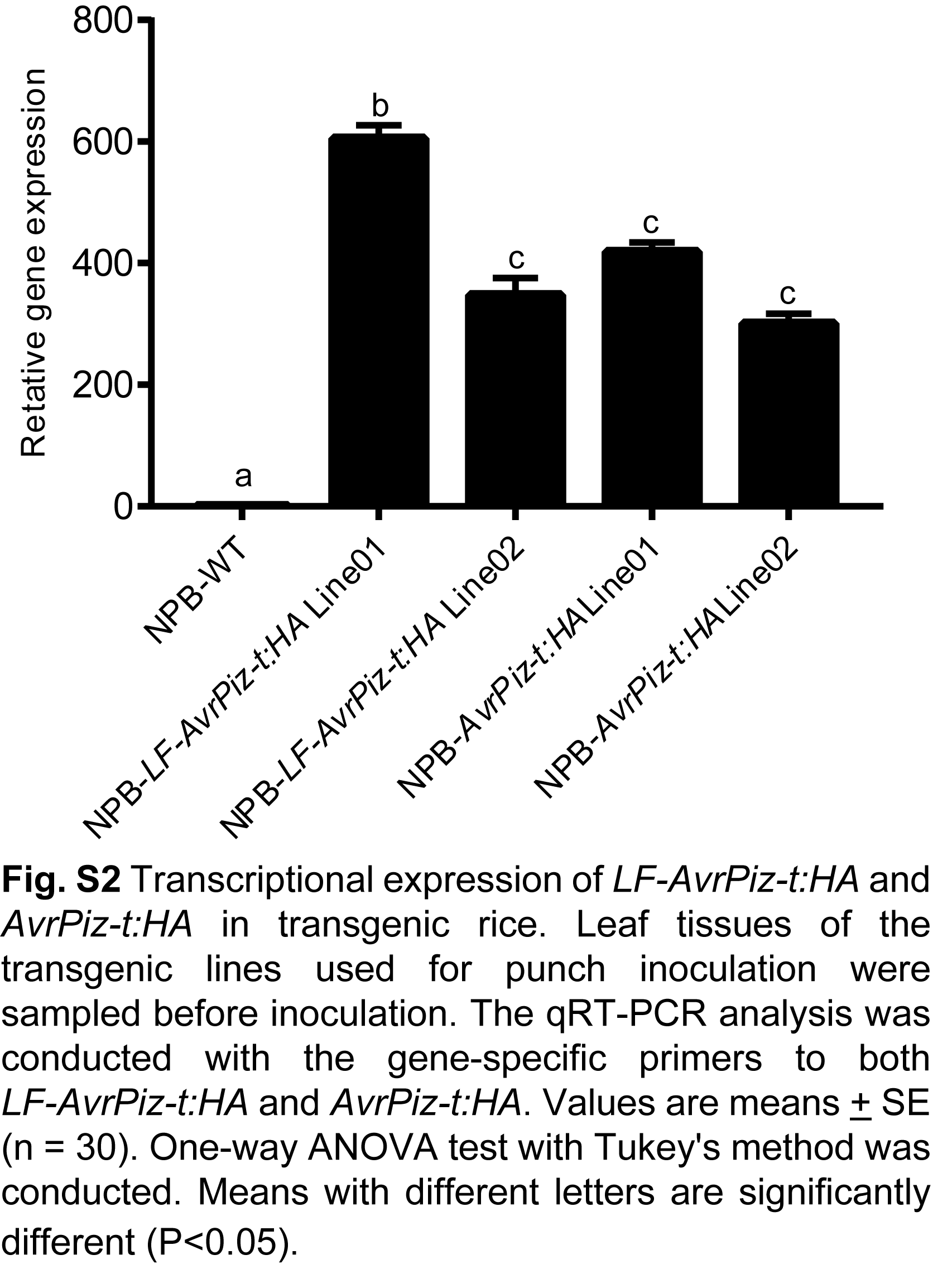

Supplement: Supplementary file 2 — Fig. S2 Transcriptional expression of LF‐AvrPiz‐t:HA and AvrPiz‐t:HA in transgenic rice. Leaf tissues of the transgenic lines used for punch inoculation were sampled before inoculation. The quantitative real‐time polymerase chain reaction (qRT‐PCR) analysis was conducted with the gene‐specific primers to both LF‐AvrPiz‐t:HA and AvrPiz‐t:HA. Values are means ± standard error (SE) (n = 30). One‐way analysis of variance (ANOVA) test with Tukey's method was conducted. Means with different letters are significantly different (P < 0.05). [file MPP-20-599-s002.tif]
