# Supplementary material for: Role of lysine residues of the Magnaporthe oryzae effector AvrPiz‐t in effector‐ and PAMP‐triggered immunity
Source: Mol Plant Pathol. 2019 Feb 8;20(4):599–608. doi: 10.1111/mpp.12779 (PMC6637882; doi:10.1111/mpp.12779)
Supplement: Supplementary file 3 — Table S1 List of plasmid constructs and primers used in this study. [file MPP-20-599-s003.docx]

Table S1: List of plasmid constructs and primers used in this study

| NO | Plasmid construct | Primer Sequence 5'-3' | Purpose |
| --- | --- | --- | --- |
| 1 | pGD-*GFP-AvrPiz-t:HA* | GCGGATCCAGCTTCGTACAATGCAAT | Transient expression in *N. benthamiana* |
|  |  | CGGTCGACCTATTGGCGCTGAGCCTG |  |
| 2 | pGD-*GFP:LF-AvrPiz-t:HA* | GCGGATCCAGCTTCGTACAATGCAAT | Transient expression in *N. benthamiana* |
|  |  | CGGTCGACCTATTGGCGCTGAGCCTG |  |
| 3 | pGD-*TAP* | ATGGTGGTCGACAACAAGTTCAAC | Negative control for transient expression in *N. benthamiana* |
|  |  | TTACCCTCCACTAGACAGTGCGCCGC |  |
| 4 | pCXUN-*GFP-AvrPiz-t:HA* | GCGGATCCAGCTTCGTACAATGCAAT | Transient expression in rice protoplast and rice transformation |
|  |  | CGGTCGACCTATTGGCGCTGAGCCTG |  |
| 5 | pCXUN-*GFP-LF-AvrPiz-t:HA* | GCGGATCCAGCTTCGTACAATGCAAT | Transient expression in rice protoplast and rice transformation |
|  |  | CGGTCGACCTATTGGCGCTGAGCCTG |  |
| 6 | pGD-*3.5xMyc:APIP10* | CTTGAATTCCATGGCGACCTCCGGCGGCG | Transient expression in *N. benthamiana* |
|  |  | AGAGGATCCTCAATACATTACAGCTTCCAT |  |
| 7 | pCX63-NP-*AvrPiz-t* | GCGGATCCAGCTTCGTACAATGCAAT | Fungal transformation |
|  |  | CGGTCGACCTATTGGCGCTGAGCCTG |  |
| 8 | pCX63-NP-*LF-AvrPiz-t* | GCGGATCCAGCTTCGTACAATGCAAT | Fungal transformation |
|  |  | CGGTCGACCTATTGGCGCTGAGCCTG |  |
| 9 | pCAMBIA1300-*AvrPiz-t-Cluc* | CGGGTACCAGCTTCGTACAATGCAATC | LCI assay |
|  |  | CGGTCGACCTATTGGCGCTGAGCCTG |  |
| 10 | pCAMBIA1300-*LF-AvrPiz-t-Cluc* | CGGGTACCAGCTTCGTACAATGCAATC | LCI assay |
|  |  | CGGTCGACCTATTGGCGCTGAGCCTG |  |
| 11 | pCAMBIA1300-*OsRBOHB-Cluc* | cgcGGTACCATGGCTGACCTGGAAGCAGG | LCI assay |
|  |  | acgcGTCGACGAAGTTCTCCTTGTGGAAATCA |  |
| 12 | pCAMBIA1300-*Nluc-OsRac1* | atttGGATCCATGAGCTCGGCGGCGGCGG | LCI assay |
|  |  | atttGTCGACCGCGAAACAAGCGCTTCCGCA |  |
