# Supplementary material for: Role of lysine residues of the Magnaporthe oryzae effector AvrPiz‐t in effector‐ and PAMP‐triggered immunity
Source: Mol Plant Pathol. 2019 Feb 8;20(4):599–608. doi: 10.1111/mpp.12779 (PMC6637882; doi:10.1111/mpp.12779)
Supplement: Supplementary file 4 — Table S2 List of primers used for quantitative real‐time polymerase chain reaction (qRT‐PCR). [file MPP-20-599-s004.docx]

Table S2: List of primers used for qRT-PCR

| NO | Plasmid and gene ID | Primer Sequence 5'-3' | Purpose |
| --- | --- | --- | --- |
| 1 | *OsPAL4*(Os02g41680) | CTACCCGCTGATGAAGAAGC | qRT-PCR primer for *OsPAL* |
|  |  | GAACCTTGTTCAGCTCCTCG |  |
| 2 | *OsNAC4*(Os01g60020) | TCCTGCCACCATTCTGAGATG | qRT-PCR primer for *OsNAC4* |
|  |  | TTGCAGAATCATGCTTGCCAG |  |
| 3 | *OsUBQ*( Os03g13170) | CGCAAGAAGAAGTGTCA | qRT-PCR primer for rice ubiquitin |
|  |  | GGGAGATAACAACGGAAGCA |  |
| 4 | *OsAPIP10* (Os02g09060) | GGGAACAAGCTGCCATTAGA | qRT-PCR primer for *OsAPIP10* |
|  |  | CTTCTGCCACGGTATTTTGG |  |
| 5 | *TAP* tag (AY436345) | CCTCCATCTCCCAAACCT | qRT-PCR primer for *TAP* tag |
|  |  | GCTCTTCCATCTGCTGCTCT |  |
| 6 | *MoPot2* (MGG_13294.6) | ACGACCCGTCTTTACTTATTTGG | qRT-PCR primer for *MoPot2* |
|  |  | AAGTAGCGTTGGTTTTGTTGGAT |  |
| 7 | *MoAvrPiz-t*(MGG_18041) | AGACACTGGGGCACGATAAG | qRT-PCR primer for *MoAvrPiz-t* |
|  |  | CCGGAGGAGAGAACATCAG |  |
